# Oxygen Variations, 2nd Edition: Integrative Insights from Cellular Models to Clinical Physiology

**DOI:** 10.3390/ijms27041863

**Published:** 2026-02-15

**Authors:** Costantino Balestra, Gerardo Bosco, Simona Mrakic-Sposta

**Affiliations:** 1 Environmental, Occupational, Aging (Integrative) Physiology Laboratory, Haute Ecole Bruxelles-Brabant (HE2B), 1160 Brussels, Belgium; 2 Anatomical Research and Clinical Studies, Vrije Universiteit Brussels (VUB), 1090 Brussels, Belgium; 3 DAN Europe Research Division (Roseto-Brussels), 1160 Brussels, Belgium; 4 Physical Activity Teaching Unit, Motor Sciences Department, Université Libre de Bruxelles (ULB), 1050 Brussels, Belgium; 5 Department of Medicine and Sciences of Aging, G. d’Annunzio University of Chieti-Pescara, 66100 Chieti, Italy; gerardo.bosco@unich.it; 6 Institute of Clinical Physiology, National Research Council (CNR), 20162 Milan, Italy; simona.mrakicsposta@cnr.it

## Introduction

As we have been studying oxygen for 250 years [1], we may think that we know it all. Admittedly, we have learned a considerable amount since the first use of the term “phlogiston”, a word coined by Jonass Johann Joachim Becher in the 17th century to refer to what we now call oxygen. Phlogiston is the neutral form of “phlogistos”, meaning “inflammable”. The word comes from “phlogizein”, meaning “put on fire, burning”, and from “phlox” (in the genitive form “phlogos”), meaning “flame”; it was the hypothetical theory of fire and was a constituent of all combustible material.

What would now be described as oxidation was thought to be the release of “phlogiston”, and the material left behind, such as ash, would be considered a “dephlogisticated” substance.

This understanding is not far from a thermodynamic perspective, in which something “on fire” has a different entropy than what remains after combustion—the so-called dephlogisticated substances. Oxygen or phlogiston is also currently considered an “epistemic object” and a number of preeminent scientists were involved in understanding it over the years, showing how much attention it still deserves [2,3,4,5].

This editorial builds upon the Special Issue “Oxygen Variations—2nd Edition” by integrating additional recent contributions addressing oxidative stress, redox regulation, and oxygen-driven adaptation across experimental, organ-specific, and clinical contexts that will propose new integrative insights from cellular models to clinical physiology [6,7].

All studies included can be interpreted through the dual lenses of hormesis, a beneficial adaptive zone lying between the “friend or foe” [8,9,10,11,12,13,14,15] nature of oxygen [16,17], and non-equilibrium thermodynamics, emphasizing oxygen variability as a structured biological stimulus rather than a static exposure [18].

1. **Experimental and Cellular Models of Oxidative Stress**

The tentative modeling of oxygen responses has been an objective for number of scientists. Sidorova et al. [19] provide a rigorous comparative framework for modeling oxidative stress in vivo using dietary, chemical, and physiological stressors in Wistar rats. From a hormetic standpoint, the study elegantly demonstrates that not all oxidative stressors are equivalent: diet-induced oxidative stress elicited the strongest redox response, characterized by concomitant increases in lipid peroxidation and antioxidant enzyme activity, while immobilization stress produced comparatively modest effects, opening up new perspectives on microgravity oxidative stress during spatial incursions.

Thermodynamically, these models represent distinct magnitudes and durations of entropy production, with dietary overload imposing a sustained metabolic disequilibrium, whereas acute stressors trigger transient adaptive responses.

This work provides a valuable platform for evaluating interventions that aim to shift oxidative stress from pathological to adaptive domains, as shown in other domains such as performance or physical training [20,21,22].

At the cellular level, Orzoł et al. [23] demonstrate that the modulation of oxidative stress can reverse premature senescence in equine adipose-derived stromal cells affected by metabolic syndrome.

Orientin treatment restored proliferation, migration, and clonogenicity while reducing senescence markers and oxidative load, paving a promising therapeutical path [24]. Within a hormetic framework, orientin appears to recalibrate redox signaling toward a low-stress adaptive zone.

Thermodynamically, the intervention reduces dissipative losses associated with chronic oxidative stress, allowing cellular energy fluxes to be reallocated toward regeneration rather than damage control.

2. **Mitochondrial Dysfunction and Redox-driven Pathology**

Pierro et al. [25] extend the discussion to clonal hematological disease, identifying oxidative stress and mitochondrial dysfunction as central drivers in myelodysplastic syndromes.

Persistent ROS (Reactive Oxygen Species) and RNS (Reactive Nitrogen Species) production destabilizes genomic integrity and promotes clonal evolution, representing a failure of hormetic containment. In thermodynamic terms, the bone marrow niche becomes trapped in a high-entropy state, where energy dissipation exceeds adaptive capacity. The review highlights redox-modulating therapies as potential tools to restore controlled signaling and re-establish a lower-entropy, functionally productive hematopoietic environment.

3. **Brain, Vascular Tone, and Neuroenergetics**

The brain constitutes a critical testbed for oxygen-driven hormesis. Salvagno et al. [26] analyze how ROS and RNS regulate cerebral vascular tone, emphasizing that species-specific redox signaling can induce vasodilation or vasoconstriction.

From a thermodynamic perspective, cerebral autoregulation emerges as a fine-tuned balance between oxygen delivery, mitochondrial efficiency, and entropy minimization in a high-demand organ.

From a therapeutical perspective, Münz et al. [27] provide experimental evidence that targeted moderate hyperoxemia improves neurological outcomes after combined subdural hematoma and hemorrhagic shock without exacerbating oxidative or nitrosative tissue damage.

This supports the concept that carefully dosed hyperoxia operates within a hormetic window, enhancing systemic organization rather than inducing oxidative collapse.

In the same direction, Damato et al. [28] further demonstrate that non-steady-state oxygen exposure produces distinct neurovascular and inflammatory responses compared to steady-state hyperoxia. Oscillating FiO_2_ induced delayed cytokine responses and modulate cortical activation, underscoring the importance of temporal oxygen variability. Thermodynamically, these oscillations represent repeated controlled perturbations that reshape neurovascular coupling and inflammatory signaling.

4. **Hyperoxia, (Relative) Hypoxia, and Systemic Adaptation**

The concept of oxy-inflammation [29] is described by Vezzoli et al. [30] in underwater activities, where divers experience alternating hypoxic and hyperoxic conditions under hyperbaric stress. The diving response exemplifies a naturally occurring hormetic system in which oxygen fluctuations, pressure, and environmental stressors jointly modulate redox and inflammatory pathways [31,32,33,34].

Changing the classical hyperbaric levels clinically used, Cannellotto et al. [35] clarify that even mild-pressure hyperbaric oxygen therapy generates sufficient mitochondrial hyperoxia to activate hypoxia-related signaling pathways through relative post-exposure hypoxia.

This apparent paradox aligns with thermodynamic principles: transient oxygen excess increases electron flux, followed by adaptive signaling that enhances angiogenesis and tissue repair. As already proposed by Salvagno et al. [36], this relative hypoxia is a potent trigger for physiological adaptations.

Using higher oxygen levels, Nesovic Ostojic et al. [37] demonstrate that hyperbaric oxygen preconditioning reduces oxidative damage and DNA strand breaks in ischemia–reperfusion acute kidney injury while upregulating HIF-1α and suppressing NF-κB.

This dual action exemplifies hormesis [38,39], where controlled oxidative signaling primes antioxidant capacity and genomic stability, allowing the system to better withstand subsequent energetic insults [40,41,42,43].

5. **Integrative Perspective and Conclusions**

Taken together, the studies included in this Special Issue and its extensions converge on a unifying concept: oxygen variability, when precisely controlled, operates as a powerful regulator of biological organization.

Hormesis provides the dose–response framework, while thermodynamics explains how transient increases in entropy production can drive reorganization toward more efficient and resilient states.

Future research should focus on defining individualized oxygen dosing strategies and identifying biomarkers that discriminate adaptive from maladaptive redox responses. It should aim to characterize the multiscale biological effects of oxygen partial pressure variations. Controlled oscillations between hypoxia, normoxia, hyperoxia, and hyperbaric hyperoxia act as non-equilibrium perturbations driving hormetic adaptation [44,45,46].

At the molecular level, transient redox signaling modulates ROS-sensitive pathways (HIF-1α, NRF2, NF-κB) and mitochondrial function.

These signals propagate to organ-level responses affecting brain energetics, vascular tone, immune regulation, and tissue repair.

Clinically, optimized oxygen variability enhances resilience, functional recovery, and therapeutic outcomes, whereas excessive or prolonged exposure may lead to maladaptive oxidative stress. It seems that too-high or too-frequent doses may offer a sub-optimal response.

6. **Conceptual Thermodynamic Framework of Oxygen-Driven Adaptation**

From a thermodynamic perspective, biological systems operate far from equilibrium and rely on continuous energy fluxes to maintain organization.

**Figure 1:**
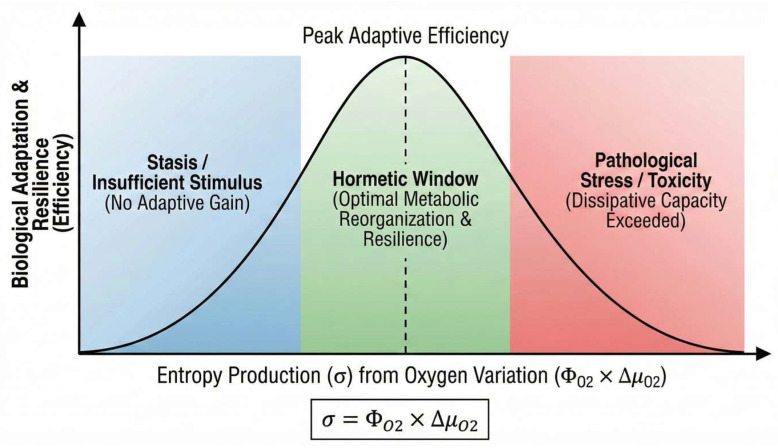
Conceptual relationship between entropy production (o), induced by variations in oxygen flux (Φ_O2_) and the chemical potential gradient (Δμ_O2_), and the adaptive biological response, illustrating the concept of the hormetic window.

Variations in oxygen availability introduce controlled perturbations that transiently increase entropy production (σ), forcing metabolic reorganization. When the perturbation magnitude remains within a hormetic window, adaptive responses emerge, leading to improved efficiency and resilience. Excessive or sustained perturbations exceed dissipative capacity [47,48] and result in non-compensated/prolonged/pathological oxidative stress (see Figure 1).

Conceptually, this relationship can be expressed in a simplified way:

σ = Φ_O2_ × Δμ_O2_

where σ represents entropy production, Φ_O2_ the oxygen flux, and Δμ_O2_ the chemical potential gradient of oxygen. Adaptive biological responses occur when σ remains within an optimal non-linear range, enabling reorganization toward lower effective entropy states following the perturbation.

The authors are confident that future research from several scientific fields will finetune “oxygen variations” [49,50] and reach specific, adaptative, hormetic, and personalized therapeutical approaches.
